# Impact of green manures of *Vernonia amygdalina* and *Chromolaena odorata* on growth, yield, mineral and proximate composition of Radish (*Raphanus sativus L*.)

**DOI:** 10.1038/s41598-019-54071-8

**Published:** 2019-11-27

**Authors:** Christopher Muyiwa Aboyeji

**Affiliations:** Department of Crop and Soil, College of Agricultural Sciences, Landmark University, Omu-Aran, Kwara State Nigeria

**Keywords:** Plant reproduction, Agroecology

## Abstract

Field experiments were carried out during the 2016 and 2017 cropping seasons at the Teaching and Research Farm, Landmark University, Omu-Aran, Kwara State (latitude 8.9°N and longitude 50°61 E.), Nigeria, to study the effect of some green manures as an alternative to inorganic fertilizer on growth, yield, mineral and proximate composition of radish. Green manure composed of leaves of *Vernonia amygdalina* and *Chromolaena odorata* and were applied as follows: 10 tonnes ha^−1^ vernonia + 0 tonnes ha^−1^ chromolaena (T_1_), 7.5 tonnes ha^−1^ vernonia + 2.5 tonnes ha^−1^ chromolaena (T_2_), 5.0 tonnes ha^−1^ vernonia + 5.0 tonnes ha^−1^ chromolaena (T_3_), 2.5 tonnes ha^−1^ Vernonia + 7.5 tonnes ha^−1^ chromolaena (T_4_), 0 tonnes ha^−1^ vernonia + 10 tonnes ha^−1^ chromolaena (T_5_) while in-organic fertilizer (NPK 20:10:10) was applied at 200 kg NPK ha^−1^ (T_6_) and there was a control plot (T_7_). The experiment was laid out in a Randomized Complete Block Design (RCBD) replicated four times. Vegetative, yield and quality parameters of radish were taken. Data collected were subjected to Analysis of Variance (ANOVA) using Statistical Analysis Software (S.A.S), 2000. Treatment means were compared using Duncan Multiple Range Test (DMRT) at 0.05 level of probability. The study showed that application of green manures increased vegetative, yield and yield parameters and were comparable with application of NPK fertilizer while there was a significant increase in the nutritional composition of radish with application of green manures when compared with NPK and control. It can therefore be concluded that application 10 tonnes ha^−1^ vernonia + 0 tonnes ha^−1^ chromolaena (T_1_) as green manure increased vegetative, yield and yield parameters while application 7.5 tonnes ha^−1^ vernonia + 2.5 tonnes ha^−1^ chromolaena (T_2_) improved radish quality.

## Introduction

Radish (*Raphanus sativus* L.), a cruciferous and nutritious root vegetable which is a good source minerals and vitamins belongs to the family Brassicaceae. It originated from Europe or Asia and is presently cultivated all over the world. Depletion in soil nutrients is considered as one of the most serious challenges facing agricultural sector in recent times resulting in significant reduction in crop growth and yield^1^. Continuous cropping on the same piece of land over time reduces the soil nutrients thereby causing a reduction in the yield of crop^2^.

In most Nigerian cultivable lands, experiments have revealed that essential plant micro- and macronutrients are far below recommended^3^. One of the best solutions of replenishing poor soils is with the use of organic fertilizers which is environmentally friendly and improves chemical and physical properties of the soil^4^. In recent years, the use of different types of organic materials and farm wastes has been documented to improve growth, yield and nutritional composition of crops when compared to the use of chemical fertilizers^5^.

Many plant materials when used as green biomass are known to improve crop productivity by increasing availability of soil N. Depending on the nature of the materials, plant materials of low C:N ratio, lignin and polyphenol decompose rapidly and are readily available sources of nutrients for crops^6^. The high cost of mineral fertilizer and its deleterious effect on the environment, rural farmers have started adopting the use of organic materials, farm residues and some farm waste to increase soil fertility and improve the physic-chemical properties of the soil. Some of the green manures that are known to improve both the physical and chemical properties of the soil are: - *Chromolaena odorata, Tithonia diversifolia, Mucuna procumbens, Gliricidia sepium, Parkia biglobosa* e.t.c. and of recent *Vernonia amygdalina*.

Vernonia, also known as bitter leaf, is an arborescent shrub that belongs to the family Asteraceae. It is found in homes, in villages as fence post and pot-herb^7^. In the derived guinea savannah of Nigeria, shrubs of vernonia are found in many farmers’ field where it is being cut down quarterly to give room for rejuvenation of fresh leaves that is mostly eaten as vegetable and for other purposes. Presently, vernonia has remained only an ornamental, fencing or wasteland plant in most parts of African countries.

Found that the use of vernonia leaves as green manure significantly improved the vegetative growth of maize^8^. In a similar experiment^9^ found that both pod yield and grain weight of cowpea increased with the application of both fresh and dry leaves of vernonia. The disposal of this biomass therefore represents loss of mineral nutrient in the soil. Laboratory analysis of the leaves therefore showed that, the leaves contained numerous nutrients that have not been adequately studied in plant nutrition.

Chromolaena has been widely used as green biomass and it is readily available to resource poor farmers. It can be found in all West African humid forest where it is known to rejuvenate the soil as a result of its high nutrient source^10^. Chromolaena has been reported to enhance and improve plant nutrient levels in the soil under its canopy through its high leaf senescence and decomposition and hence improve the physical and chemical properties of the soil^11^. The observation made by^12^, showed that chromolaena when incorporated into the soil can increase amounts of nitrogen, phosphorus, potassium, calcium, magnesium and C/N ratio but not the soil pH.

Organic manures which are environmentally friendly have been found to be a good source of nutrients in rejuvenating poor soils by improving the organic matter and the physical and chemical properties of the soil^4,13^. Recently, advocacy for organic farming and produce is on the increase in the agricultural sector nationwide. This can be achieved by providing nutrients in correct quantity and proportions in such a way that it will benefit the environment and the soil in particular^14^.

Despite the call for organically produced foods by the National Organic Standard Board (NOSB) of the United States^15^, the result of the laboratory analysis of vernonia leaves which revealed that it contained numerous nutrients and that the leaves are readily available to poor resource farmers, there is little information on its potential in supplying crop nutrients and its ability in enhancing the physical and chemical characteristics of the soil, therefore the study was initiated to determine and compare the effect of sole and combined application of vernonia and chromolaena on growth, yield and nutritional value of radish root. Based on this objective, it was hypothesized that growth, yield and nutritional composition of radish root would react differently to sole and combined application fresh biomass of vernonia and chromolaena. Studies were therefore conducted to validate this hypothesis; which combinations of these treatments have greater effect?

## Results

### Soil physical and chemical properties prior to planting (0–15 cm) in 2016 and 2017

The pre-planting soil analysis for the two years is as shown in Table 1. The pH of the soil was moderately acidic, the nitrogen content was very low, the available Phosphorus and exchangeable K were at moderate while the exchangeable Na, Ca, and Mg are all suitable. The organic Carbon and Organic matter were also low. The soil is high in sand with relatively low values in both silt and clay; hence the textural class is Sandy loam.

**Table 1:** Soil physical and chemical properties prior to planting (0–15 cm).

Values			Values		
Parameter	2016	2017	Parameter	2016	2017
Sand (%)	76.00	76.22	Exchangeable bases		
Silt (%)	13.00	12.58	K (cmol/kg)	0.13	0.15
Clay (%)	11.00	11.20	Na (cmol/kg)	0.66	0.59
Textural class	Sandy loam	Sandy loam	Ca (cmol/kg)	3.17	3.18
pH (H_2_O) 1:1	5.36	5.22	Mg (cmol/kg)	0.55	0.60
Total N (%)	0.16	0.19	ECEC (cmol/kg)	4.51	4.52
O.M (%)	2.27	2.30	Available phosphorus (mg/kg)	9.60	9.65
O.C (%)	1.32	1.36	Zn (mg/kg)	0.45	0.39

### Meteorological data of the experimental site for 2016 and 2017

The meteorological data for the periods of the experiment is as shown in Table 2. The mean rainfall for 2016 cropping season was higher than that of 2017 cropping season.

**Table 2:** Meteorological data for 2016 and 2017.

	Relative humidity (%)		Rainfall (mm)		Temperature (°C)	
	2016	2017	2016	2017	2016	2017
June	88.29	87.90	172.47	172.72	27.32	27.32
July	91.14	86.20	140.97	71.12	25.62	26.75
Total	179.43	174.10	313.44	243.84	52.94	54.07
Mean	89.72	87.05	156.72	121.92	26.47	27.04

### Analysis of the chemical composition of vernonia and chromolaena

Table 3 shows the laboratory result of the chemical composition of vernonia and chromolaena leaves. It was observed that nitrogen, phosphorus, magnesium and organic carbon content of vernonia was higher than that of chromolaena while values for potassium, calcium and C:N for chromolaena were higher than that of vernonia, though values for calcium for the two green manures were slightly similar.

**Table 3:** Chemical composition of vernonia and chromolaena.

	N (%)	P (%)	K (%)	Mg (%)	Ca (%)	O.C (%)	C:N
*V. amygdalina*	4.43a	1.95a	0.62b	0.97a	1.52a	50.5a	11.4b
*C. odorata*	3.20b	1.65b	0.85a	0.62b	1.55a	46.6b	14.5a

Means in a column under any given treatment followed by the same letter(s) do not differ significantly at 0.05 level of probability using the Duncan Multiple Range Test (DMRT). Values of the parameters were obtained from analysis of fresh biomass of vernonia and chromolaena.

### Effect of vernonia and chromolaena leaves as green manures and NPK fertilizer on plant height and number of leaves of radish

The effect of vernonia and chromolaena leaves on plant height and number of leaves of radish is as shown in Table 4. Application of NPK fertilizer (T_6_) resulted in taller plants which was similar to the application 10 tonnes ha^−1^ vernonia + 0 tonnes ha^−1^ chromolaena (T_1_) in both years and 7.5 tonnes ha^−1^ vernonia + 2.5 tonnes ha^−1^ chromolaena (T_2_) in 2016. There was no significant difference in the application of other treatments except the control (T_7_) which gave the least value for plant height.

**Table 4:** Effect of vernonia and chromolaena leaves as green manures and NPK fertilizer on plant height and number of leaves of radish in 2016 and 2017 cropping seasons.

Treatments	Plant height (cm) at 4 WAS		Number of leaves at 4 WAS	
	2016	2017	2016	2017
10 V + 0 C (T_1_)	16.67a	16.25a	8.33a	7.50a
7.5 V + 2.5 C (T_2_)	16.36a	15.00b	7.16b	6.85a
5.0 V + 5.0 C (T_3_)	13.50b	12.30c	7.00b	6.52a
2.5 V + 7.5 C (T_4_)	13.33b	11.10c	6.70b	6.10a
0 V + 10 C (T_5_)	12.50b	10.90c	6.86b	6.12a
N.P.K (T_6_)	17.83a	16.95a	9.03a	8.75a
Control (T_7_)	8.73c	8.20d	5.13c	4.80b

Means in a column under any given treatment followed by the same letter(s) do not differ significantly at 0.05 level of probability using the Duncan Multiple Range Test (DMRT).

Treatment dose per ha: -V = tonnes ha^−1^ C = tonnes ha^−1^ NPK = kg ha^−1^

V = Vernonia C = Chromolaena.

Compared with the control (T_7_) which gave the least number of leaves in 2016 and 2017 cropping seasons, more number of leaves was observed with the application of NPK (T_6_). The values were statistically similar with other treatment values in 2017 and 10 tonnes ha^−1^ vernonia + 0 tonnes ha^−1^ chromolaena (T_1_) alone in 2016.

### Effect of vernonia and chromolaena leaves as green manures and NPK fertilizer on yield and yield attributes of radish (*Raphanus sativus* L.)

**Figure 1:**
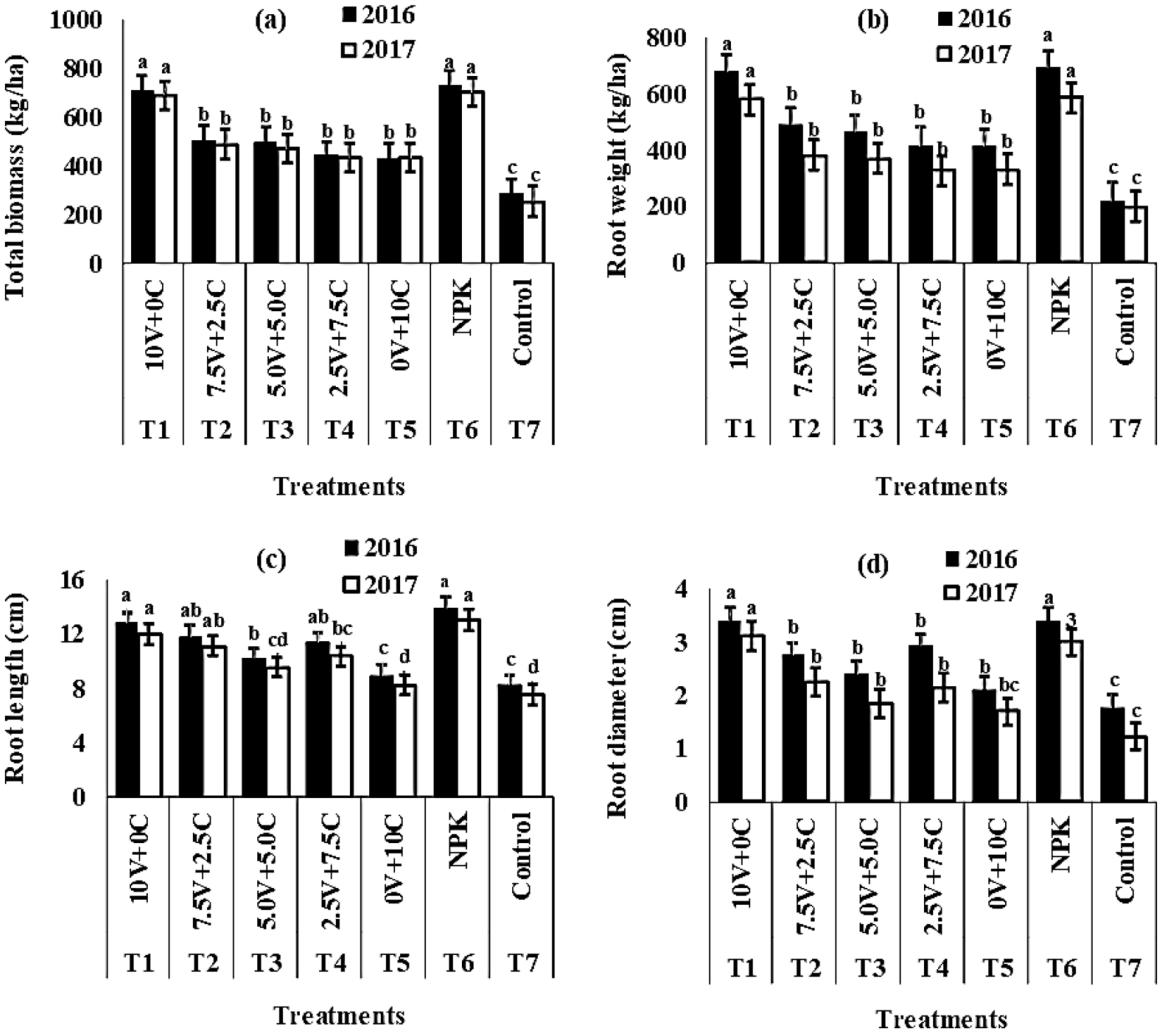
Effect of vernonia and chromolaena leaves as green manures and NPK fertilizer on **(a)** total biomass, **(b)** root weight, **(c)** root length and **(d)** root diameter of radish (*Raphanus sativus* L.) in 2016 and 2017. Vertical bars show standard errors; bars marked with different letters show means significantly different at 5% level using Duncan’s multiple range test. Values of the parameters were obtained from analysis of fresh biomass of vernonia and chromolaena. T1–T7 were used to distinguish treatments. Treatment dose per ha: - V = tonnes ha^−1^ C = tonnes ha^−1^ NPK = kg ha^−1^. V = Vernonia C = Chromolaena.

The significant influence of NPK fertilizer (T_6_) and 10 tonnes ha^−1^ vernonia + 0 tonnes ha^−1^ chromolaena (T_1_) on yield and yield attributes of radish (Fig. 1a–d) was well evidenced in the present study. When compared with the control (T_7_) which gave the least values for yield and all its attributes in both years, application of NPK fertilizer (T_6_) and 10 tonnes ha^−1^ vernonia + 0 tonnes ha^−1^ chromolaena (T_1_) significantly increased values for all the parameters. Application of 7.5 tonnes ha^−1^ vernonia + 2.5 tonnes ha^−1^ chromolaena (T_2_), 5.0 tonnes ha^−1^ vernonia + 5.0 tonnes ha^−1^ chromolaena (T_3_), 2.5 tonnes ha^−1^ vernonia + 7.5 tonnes ha^−1^ chromolaena (T_4_) and 0 tonnes ha^−1^ vernonia + 10 tonnes ha^−1^ chromolaena (T_5_) resulted in varying and non-significant values for yield and its attributes.

### Effect of varying levels of vernonia and chromolaena as green manures and NPK fertilizer on the proximate composition of radish root

**Figure 2:**
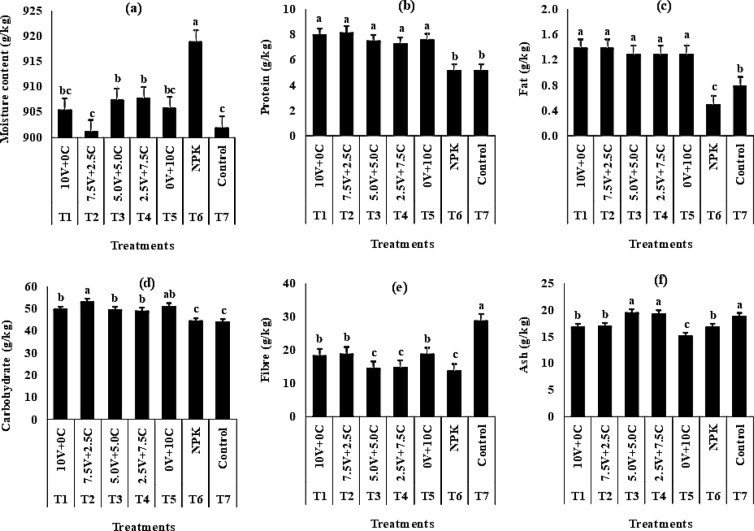
Effect of varying levels of vernonia and chromolaena as green manures and NPK fertilizer on **(a)** moisture **(b)** protein **(c)** fat **(d)** carbohydrate **(e)** fibre and **(f)** ash content of radish root (2016 and 2017 pooled data). Vertical bars show standard errors; bars marked with different letters show means significantly different at 5% level using Duncan’s multiple range test. Values of the parameters were obtained from analysis of fresh biomass of vernonia and chromolaena. T1–T7 were used to distinguish treatments. Treatment dose per ha: - V = tonnes ha^−1^ C = tonnes ha^−1^ NPK = kg ha^−1^ V = Vernonia C = Chromolaena.

Application of NPK fertilizer, sole and combined green manures significantly influenced proximate composition of radish (Fig. 2a–f). Compared with the control (T_7_), application of NPK (T_6_) significantly increased moisture content (M.C) while sole and combined application of green manures resulted in varying but statistically similar values. Percentage protein, fat and carbohydrate (CHO) increased with application of 10 tonnes ha^−1^ vernonia + 0 tonnes ha^−1^ chromolaena (T_1_) and 7.5 tonnes ha^−1^ vernonia + 2.5 tonnes ha^−1^ chromolaena (T_2_) though the values were not significant with percentage fat when other green manures applied. Application of NPK (T_6_) and control significantly reduced values for protein, fat and CHO. Control produced higher values for fibre and ash while application of sole and combined green manures gave statistically similar values.

### Effect of varying levels of vernonia and chromolaena as green manures and NPK fertilizer on vitamin C, and mineral content of radish root

Compared with application of NPK fertilizer and the control which gave lower but similar values for vitamin C, application of green manures significantly increased the vitamin C content of radish with 10 tonnes ha^−1^ vernonia + tonnes ha^−1^ chromolaena (T_1_) and 7.5 tonnes ha^−1^ vernonia + 2.5 tonnes ha^−1^ chromolaena (T_2_) having higher and statistically similar values (Table 5).

**Table 5:** Effect of varying levels of vernonia and chromolaena as green manures and NPK fertilizer on vitamin C, and mineral content of radish root (2016 and 2017 pooled data).

Treatments	Vit. C	Ca	P	K	Mg
**mg/100 g**					
10 V + 0 C (T_1_)	19.32a	26.85a	25.46a	273.00c	0.72a
7.5 V + 2.5 C (T_2_)	19.40a	27.55a	25.50a	275.65a	0.73a
5.0 V + 5.0 C (T_3_)	17.96b	27.53a	24.33b	274.10b	0.69b
2.5 V + 7.5 C (T_4_)	17.95b	27.50a	24.05b	274.62b	0.69b
0 V + 10 C (T_5_)	18.00b	27.60a	24.10b	274.54b	0.68b
N.P.K (T_6_)	16.20c	24.63b	25.42a	275.36a	0.65c
Control (T_7_)	16.55c	23.95b	21.56c	270.00d	0.50d

Means in a column under any given treatment followed by the same letter(s) do not differ significantly at 0.05 level of probability using the Duncan Multiple Range Test (DMRT). Values of the parameters were obtained from analysis of fresh biomass of vernonia and chromolaena. Treatment dose per ha: - V = tonnes ha^−1^ C = tonnes ha^−1^ NPK = kg ha^−1^ V = Vernonia C = Chromolaena.

Mineral composition of radish root also varied significantly with the application of green manures and NPK fertilizer (Table 5). Calcium content of radish root increased significantly with the application of green manures when compared with application NPK (T_6_) fertilizer and control (T_7_) which gave statistically similar but lower values. Application of 7.5 tonnes ha^−1^ vernonia + 2.5 tonnes ha^−1^ chromolaena (T_2_) significantly increased values for P. K and Mg though statistically similar with the application of NPK (T_6_) only in P and K. There was no significant difference in P, K and Mg values when 5.0 tonnes ha^−1^ vernonia + 5.0 tonnes ha^−1^ chromolaena (T_3_), 2.5 tonnes ha^−1^ vernonia + 7.5 tonnes ha^−1^ chromolaena (T_4_) and 0 tonnes ha^−1^ vernonia + 10 tonnes ha^−1^ chromolaena (T_5_) were applied. Control plots showed least values for P, K and Mg.

## Discussion

Laboratory determination of the nutrient elements contained in the leaves of vernonia and chromolaena revealed that they both contained mineral elements in varying proportions suitable for the cultivation of radish. There was quick response of radish when both the green manures and inorganic fertilizer were applied in both years of the experiments. The response of radish to the amendments could be as a result of continuous cropping and the type of dominant weeds that were found on the experimental field which have depleted most of the essential nutrient elements required for the cultivation of radish.

Vegetative parameters were significantly affected by the application of green manures of vernonia and chromolaena. Plant height and number of leaves were higher in plants treated with NPK fertilizer which was comparable with plots treated with 10 tonnes ha^−1^ vernonia + 0 tonnes ha^−1^ chromolaena (T_1_) alone and 7.5 tonnes ha^−1^ vernonia + 2.5 tonnes ha^−1^ chromolaena (T_2_). Increase in the rate of application of vernonia was also found to increase values for plant height and number of leaves. The increase in the vegetative parameters as a result of application of green manures could be attributed to the chemical composition of the green manures which increased soil availability of the nutrients most especially Mg which is part of chlorophyll molecule located in the thylakoid membrane of the leaves that are essential for increased photosynthesis^16^. Also found that incorporation of green manures increased soil availability of OM, N, P, K and Mg. Increase in plant height and number of leaves of radish could also be adduced to high moisture content and low C:N ratio contained in the leaves of vernonia and chromolaena leading to early mineralization and fast release of nutrients^17^. Similar result was obtained by^18^, who reported that the application of organic fertilizers to soil supply plant nutrients for increased plant height and more leaves of shallot.

Results of this study showed that application of NPK (T_6_) and 7.5 tonnes ha^−1^ vernonia + 2.5 tonnes ha^−1^ chromolaena (T_2_) increased vegetative and yield parameters of radish. This could be as a result of nitrogen that was present in both amendments which helped to increase plant growth and development. Nitrogen is frequently the most limiting essential nutrient in natural ecosystems^19^. The increase in these parameters as a result of application of NPK fertilizer could be due to early availability of the nutrients culminating into enhanced nutrient uptake and hence faster growth. This is in line with the work of some researchers^20^ who found that while nutrient supplied by in-organic fertilizer was readily available, the nutrient supplied by organic fertilizer was released slowly.

Incorporation of 10 tonnes ha^−1^ vernonia + 0 tonnes ha^−1^ chromolaena (T_1_) increased yield and yield parameters (total biomass, root length, root weight and root diameter) of radish and it was comparable with application of NPK fertilizer.

Taller plants and more number of leaves may have contributed to increased yield and yield parameters. This is because plant height and increased number of leaves could accumulate more environmental resources thereby transporting them to the sinks (roots) of the plant. Similar result was by^21^ where they found that biomass yield, root weight, root length and root diameter of radish increased with application of tithonia up to 10 tonnes ha^−1^. Increase plant vigour and yield as a result of application of green manures could be adduced to its ability to improve soil physical and chemical properties^22^.

Sole application of vernonia performed better than sole application of chromolaena in terms of vegetative growth, yield and yield parameters and in the same vein, results showed that, as the level of vernonia increased in the combination of the green manures, the better the performance of radish. This could be as a result of the inherent chemical composition of vernonia. It could also be as a result of its high organic carbon when compared with chromolaena. High organic matter in the soil as a result of application of green manure must have increased microbial activities in the soil thereby increasing soil physical characteristics and porosity. Presence of green manures increased activities of beneficial soil fauna and organic matter decomposition, which could lead to enhanced soil porosity and reduced soil bulk density^23^.

Result of the study also showed that the rate of mineralization of vernonia and chromolaena leaves varied significantly with vernonia having higher degree of mineralization. This could be attributed to higher N value and lower C:N ratio of vernonia^24^. In their experiments found that rate of litter decomposition depends on the C:N ratio and/or nitrogen content of the leaf litter. There was a consistent increase in growth, yield and yield parameters of radish in 2016. This could be as a result of higher rainfall experienced during the weeks of the experiment which facilitated decomposition of the green manures thereby releasing its nutrients for plant use.

Phosphorus fertilizer facilitates the production of more roots and increased number of branches. Both the green manure and NPK contained suitable quantity of phosphorus. Total biomass and root yield increased with application of 10 t ha^−1^ vernonia + 0 tonnes ha^−1^ chromolaena (T_1_) and NPK (T_6_). Increase in the values for these parameters could be as a result of one of the functions of phosphorus in facilitating the production of more roots or that the soil was low in the nutrient. Like nitrogen, phosphorous is an essential constituent of the genetic material and augments cell division^22^.

Application of 10 tonnes ha^−1^ vernonia + 0 tonnes ha^−1^ chromolaena (T_1_) competes favorably with NPK (T_6_). This could be as a result of presence of calcium in vernonia which function in plant root and tip elongation^25^. Reported that soil applied with tithonia enhanced stem girth better than NPK applied soils.

Application of vernonia and chromolaena leaves as green manures either as sole or in combination with each other increased the proximate composition of radish root. This increase could be as a result of availability of sufficient nutrient for plant uptake which was later translocated into the appropriate sources where they were partitioned into their respective sinks^26^. Concluded that compost application to nutrient limiting soil improved quality and quantity of both biomass and proximate constituents of maize.

Sole and combined application of green manures increased vitamin C content and Mg of radish roots with application of 10 tonnes ha^−1^ vernonia + 0 tonnes ha^−1^ chromolaena (T_1_) and 7.5 tonnes ha^−1^ vernonia + 2.5 tonnes ha^−1^ chromolaena (T_2_) having higher values when compared with application of NPK (T_6_) and the control (T_7_). This could also be attributed to increased availability of nutrients in soil as a result of the mineralization of the manures leading to increased uptake by the plants. Similar result was by^27^ who found that conventional production system of lettuce resulted in lower vitamin C content^28^. Also reported that the vegetables produced under organic systems frequently had higher contents of vitamin C, when compared with those produced conventionally.

Laboratory analysis of vernonia and chromolaena showed that there was no significant difference in Ca values of both leaves. This could possibly explain why Ca values of radish root did not differ significantly among sole and combined application of green manures. Application of green manures and NPK fertilizer increased P and K contents of radish roots significantly compared with the control. This could be as a result of reasonable amount of the nutrients present in the leaves of the green manures and the in-organic fertilizer used for the study. The reason for low level of P, K and Mg in radish root in the control plots could be that the soil has been depleted of the nutrients as a result of continuous cropping and the type of dominant weeds found on the field before the start of the experiment.

## Conclusion

The observed response of radish to sole and combined application of vernonia and chromolaena suggests that they can be used as an alternative to in-organic fertilizer because they contained mineral nutrients in varying proportions and are capable to improved soil physical and chemical properties. It can therefore be concluded that while 10 tonnes ha^−1^ vernonia + 0 tonnes ha^−1^ chromolaena (T_1_) as green manure is recommended for increased vegetative, yield and yield parameters, 7.5 tonnes ha^−1^ vernonia + 2.5 tonnes ha^−1^ chromolaena (T_2_) is recommended for improved vitamin C, protein, carbohydrate and mineral content of radish.

## Materials and Methods

The experiment was conducted during 2016 and 2017 cropping seasons at the Teaching and Research Farm of Landmark University, Omu-Aran, Kwara State (lat8^0^ 9′ N and long 5^0^ 61′ E.) located in the derived savanna ecological zone of Nigeria to study the effect of fresh leaves of vernonia and chromolaena as green manures on the growth, yield and nutritional composition of radish. The ecological zone has an annual rainfall pattern which extends between the months of April and October and it ranges between 600–1500 mm, with peak rain in May-June and September-October, while the dry season is between November and March. The experimental sites had been under continuous cropping for more than five years while the dominant weeds were itch grass (*Rottboellia cochinchinensis* (Lour) Claton), goose grass (*Eleusine indica* (L.) Gaertn), sour millet (*Echinochloa colona* (L.) Link), Milkweed (*Euphorbia heterophylla* L) and goat weed (*Ageratum conyzoides* L.).

Pre-cropping soil samples were collected from each plot during the cropping seasons and bulk to determine the physicochemical properties of the soil. Mechanical land preparation was adopted using the tractor drawn disc plough and harrow. The land was ploughed once and harrowed twice to give a well pulverized soil. Thereafter the field layout was carried out to mark out the appropriate number of treatment plots. The size for each bed was (2 m × 2 m) = 4m^2^ and the total net plot size used for the experiment was (28 m × 4 m) = 122 m^2^.

Green tender leaves and stem of vernonia were collected from the Research Farm of the Biofuels Alternatives and Renewable Energy Ltd, Edidi, Kwara State Nigeria while chromolaena was collected on a fallow field at Landmark University Teaching and Research Farm, Omu-Aran. The leaves were picked from the stem, chopped into pieces with a sharp knife for easier decomposition, weighed and applied to a depth of about 10 cm two weeks before seed sowing using hand held hoes according to the layout.

The followings are the treatment combinations which were applied as green biomass two weeks before seed sowing to give room for decomposition: − 10 tonnes ha^−1^ vernonia + 0 tonnes ha^−1^ chromolaena (T_1_), 7.5 tonnes ha^−1^ vernonia + 2.5 tonnes ha^−1^ chromolaena (T_2_), 5.0 tonnes ha^−1^ vernonia + 5.0 tonnes ha^−1^ chromolaena (T_3_), 2.5 tonnes ha^−1^ vernonia + 7.5 tonnes ha^−1^ chromolaena (T_4_), 0 tonnes ha^−1^ vernonia + 10 tonnes ha^−1^ chromolaena (T_5_), 200 kg NPK ha^−1^ (T_6_) and no fertilizer/green manure control (T_7_). There were seven (7) plots in each replicates, making a total of twenty eight (28) experimental units. Treatments were laid out in Randomised Complete Block Design and replicated four times.

The variety of radish used for the experiment was ‘French breakfast’. At two weeks after incorporation of green manures, seventy two seeds were sown second week of June during the experimental years at a depth of 5 cm on a prepared 2 m by 2 m bed at two seeds per hole with 30 cm by 30 cm inter and intra-row spacing. Plants were thinned to one plant per stand one week after sowing leaving a total number of 36 plants per bed i. e 11, 111 stands ha^−1^. Inorganic fertilizer (NPK 20:10:10) was manually applied to the assigned plots at the rate of 200 kg NPK ha^−1^ (equivalent to 40 kg N ha^−1^, 20 kg P ha^−1^ and 20 kg K ha^−1^) two weeks after sowing by side placement 5–8 cm away from the base of the plant.

Manual weeding was done 2 weeks after sowing (WAS) and it was supplemented with hand-picking of weeds 4 WAS. Radish roots were harvested 6 WAS by hand pulling the roots from the ground. The following vegetative, yield and yield component parameters were taken in both years of the experiment, plant height, number of leaves, total biomass, root weight, root length, root diameter while proximate, minerals and vitamin C values were collected for the roots as post-harvest parameters.

### Laboratory analysis of vernonia and chromolaena leaves and determination of minerals and Vitamin C content of radish roots

Samples of vernonia, chromolaena and radish root were collected fresh and oven-dried for 24 h at 80^o^C and grinded in a Willey mill. Leaf samples of vernonia and chromolaena were analysed for leaf N, P, K, Ca and Mg while radish root samples were analysed for P, K, Ca and Mg as described by^29^. Leaf N was determined by the micro-Kjeldahl digestion method. Ground samples were digested with nitric-perchloric-sulphuric acid mixture for the determination of P, K, Ca and Mg. Phosphorus was determined colorimetrically using the vanadomolybdate method, K was determined using a flame photometer and Ca and Mg were determined by the EDTA titration method^30^. The percentage of organic carbon in the sample was determined by the Walkley and Black procedure using the dichromate wet oxidation method^31^. Sample pH was determined by using a soil–water medium at a ratio of 1:2 using Jenway digital electronic pH meter model 3520^32^. Vitamin C content of radish root was determined by using the indophenol dye method^33^.

### Proximate analysis of radish roots

Proximate analysis of radish root was carried out at the central laboratory, Landmark University, Nigeria. Root samples were grinded and analyzed for their proximate composition under the Parten D analyzer. The root samples were analyzed to determine the Moisture content, crude fiber content, carbohydrate content, protein content, ash content and the Fat content.

### Statistical analysis

Data collected were subjected to statistical analyses of variance (ANOVA) using statistical Analysis Software^34^. The significant treatment means were compared using Duncan Multiple Range Test (DMRT) at 0.05 level of probability.
